# WTAP Contributes to Periodontitis Pathogenesis by Promoting PDLSC Senescence and Impairing Osteogenic Differentiation via m6A‐Dependent Regulation of TP53BP1

**DOI:** 10.1002/iid3.70335

**Published:** 2026-02-05

**Authors:** Menglin Xiong, Tingting Wang, Yuan Liu

**Affiliations:** ^1^ Department of Cariology and Endodontics School/Hospital of Stomatology, The First Affiliated Hospital of Xinjiang Medical University Urumqi Xinjiang China

**Keywords:** N6‐methyladenosine, osteogenic differentiation, periodontitis, TP53BP1, WTAP

## Abstract

**Background:**

Periodontitis, a chronic inflammatory disease, represents the primary cause of tooth loss in Chinese adults. Wilms tumor 1‐associating protein (WTAP) is a key component of the N6‐methyladenosine (m6A) methyltransferase complex, and has an unclear role in periodontitis pathogenesis, particularly concerning its regulatory functions in periodontal ligament stem cells (PDLSCs).

**Methods:**

The target gene was identified through the GES260558 dataset and Genecards database. Gene expression was measured using reverse transcription‐quantitative PCR (RT‐qPCR) and western blot. Periodontitis‐derived PDLSCs (P‐PDLSCs) were isolated and identified by alkaline phosphatase (ALP) staining, oil red O staining, and flow cytometry. Malondialdehyde (MDA), superoxide dismutase (SOD), and reactive oxygen species (ROS) levels, γ‐H2AX and SA‐β‐gal positive cells, and the expression of p53 and p16 were applied to reflect oxidative stress and cell senescence. Osteogenic differentiation was assessed by ALP activity, alizarin red S (ARS) staining, and related gene expression. The m6A‐dependent regulation of tumor protein p53 binding protein 1 (TP53BP1) mRNA by WTAP was confirmed using methylated RNA immunoprecipitation (MeRIP), RNA immunoprecipitation (RIP), and Actinomycin D (Act D) assays.

**Results:**

WTAP was identified as a candidate gene that was upregulated in periodontitis gingival tissues. Isolated P‐PDLSCs retained normal multilineage differentiation potential. WTAP knockdown significantly reduced senescence and oxidative stress in P‐PDLSCs while enhancing osteogenic differentiation. Mechanistically, WTAP mediated the m6A modification of TP53BP1 mRNA, and the effects of WTAP on P‐PDLSC senescence, oxidative stress, and osteogenic differentiation were dependent on TP53BP1.

**Conclusion:**

The WTAP/TP53BP1 axis impairs periodontal tissue regeneration by promoting P‐PDLSC senescence and suppressing osteogenic differentiation in an m6A‐dependent manner, revealing a new cellular‐level target for treating periodontitis.

## 1 Introduction

Periodontitis is a prevalent immune‐inflammatory disease characterized by the progressive destruction of periodontal ligaments and alveolar bones, primarily driven by the host's dysregulated immune response to bacterial [1]. This chronic inflammatory milieu, rich in pro‐inflammatory cytokines, not only directly damages periodontal tissues but also profoundly impairs the function of resident stem cells, thereby hampering tissue regeneration [2]. Its high prevalence has a great impact on patients' lives, contributing significantly to the global burden of disease [3].

Periodontal ligament stem cells (PDLSCs) are key mesenchymal stem cells responsible for periodontal homeostasis and regeneration [4, 5, 6]. However, in the setting of periodontitis, the inflammatory microenvironment induces PDLSC dysfunction, leading to cellular senescence and diminished osteogenic potential—a critical barrier to healing [7]. Therefore, understanding the mechanisms that disrupt PDLSC function under inflammatory stress is central to developing novel regenerative strategies.

In recent years, epigenetics, and specifically N6‐methyladenosine (m6A) RNA modification, has emerged as a pivotal layer of regulation in immune and inflammatory responses [8]. For instance, m6A modifications can determine the fate of inflammatory transcripts and influence immune cell activation [9]. The m6A process is dynamic and reversible, regulated by writers, erasers, and readers [10]. Wilms tumor 1‐associating protein (WTAP), a key component of the writer complex, has been implicated in fine‐tuning gene expression in various diseases, including cancer [11] and inflammatory conditions like arthritis [12]. Notably, the study by Li *et al*. highlighted the role of WTAP in macrophage M1 polarization within periodontitis [13], underscoring its involvement in the local immune landscape. However, its function in regulating the core cellular unit of regeneration—the PDLSC—within this inflammatory context remains entirely unexplored.

Tumor protein p53 binding protein 1 (TP53BP1) is a DNA damage response protein [14] that also promotes PDLSC senescence in inflammatory environments [15]. While its regulation by alternative splicing is known [15], whether it is subject to post‐transcriptional control by epitranscriptomic mechanisms like m6A, and how this integrates with the inflammatory signaling in periodontitis, is unknown.

This research hypothesizes that the inflammatory microenvironment in periodontitis hijacks the m6A machinery to drive PDLSC dysfunction, aiming to elucidate the mechanism of WTAP's involvement in periodontitis, focusing on its regulation of PDLSC senescence and osteogenic differentiation, a key cellular event *in vitro*. This work positions m6A modification as a critical link between inflammation and regeneration, offering a new therapeutic perspective that extends beyond the conventional antimicrobial approach to periodontitis management.

## 2 Materials and Methods

### 2.1 Data Collection and Analysis

Differentially expressed genes (DEGs) were analyzed from the GSE260558 dataset (https://www.ncbi.nlm.nih.gov/search/all/?term=GSE260558) downloaded from the Gene Expression Omnibus (GEO) database. GEO2R (https://www.ncbi.nlm.nih.gov/geo/geo2r/) was used to recognize DEGs based on the standard of *p* < 0.05 and |log2FC | ≥ 1. The dataset comprised two groups: H‐PDLSCs (PDLSCs cultured in normal medium) and I‐PDLSCs (PDLSCs cultured in inflammatory cytokine‐induced medium), representing homeostatic and inflammatory conditions, respectively. The volcano plot of the DEGs was generated employing the Xiantao academic website (https://www.xiantaozi.com/).

To identify target genes, the DEGs identified from the GSE260558 dataset and the senescence‐related genes obtained from the Genecards database (https://www.genecards.org/) were intersected, retaining only protein‐coding genes with a relevance score ≥ 10 (median threshold).

### 2.2 Human Samples

Gingival tissues were collected from patients undergoing tooth extraction for orthodontic reasons (control, *N* = 20) or due to periodontitis (periodontitis, *N* = 28). All procedures met a criterion of School/Hospital of Stomatology, The First Affiliated Hospital of Xinjiang Medical University Ethics Committee. All patients signed informed consent prior to sample collection. The consent process involved a detailed explanation of the study's purpose, procedures, potential risks, and benefits, assuring participants of confidentiality and their right to withdraw at any time.

To standardize tissue collection, all procedures were performed by the same experienced periodontist. Periodontitis was diagnosed based on the following clinical parameters: probing depth (PD) ≥ 5 mm, clinical attachment loss (CAL) ≥ 3 mm, and radiographic evidence of alveolar bone loss. Control tissues were obtained from periodontally healthy individuals (PD ≤ 3 mm, no CAL or bleeding on probing) with intact periodontium.

To control for potential confounders, patients with a history of systemic diseases (like diabetes), those who were smokers, had undergone periodontal therapy in the last 6 months, or were on medications known to affect periodontal status (such as phenytoin and cyclosporine) were eliminated from the study. The gender distribution and average age of the donors were as follows: Control group: 11 males/9 females mean age 45.6 ± 8.3 years; Periodontitis group: 16 males/12 females, mean age 43.1 ± 9.5 years.

### 2.3 Reverse Transcription‐Quantitative PCR (RT‐qPCR)

The RNA was obtained utilizing the RNAsimple Total RNA Kit (DP419, Tiangen, Beijing, China). Reverse transcription was conducted with the BeyoRT II First Strand cDNA Synthesis Kit with gDNA Eraser (D7170M, Beyotime, Shanghai, China) under the following conditions: Genomic DNA removal at 37°C for 2 min, reverse transcription at 42°C for 60 min, and enzyme inactivation at 80°C for 10 min. The qPCR reaction system was prepared by mixing cDNA with SYBR Green Ⅰ (D7405, Beyotime), forward and reverse primers, PCR Buffer (with Mg^2+^), dNTPs (2.5 mM each), Taq DNA Polymerase (5 U/μL), and ultrapure water. qPCR was conducted on the Archimed R4 fluorescent quantitative PCR system (Rocgene, Beijing, China). β‐actin was used as the reference gene, and relative gene expression was quantified by exploiting the 2^−^^ΔΔCt^ method. The primer sequences were as follows: WTAP: Forward (5′–3′) AGGGCAACACAACCGAAGAT, Reverse (5′–3′) ACCCCGCACTGAGTTGATTT; TP53BP1: Forward (5′–3′) ATGGACCCTACTGGAAGTCAG, Reverse (5′–3′) TTTCTTTGTGCGTCTGGAGATT; β‐actin: Forward (5′–3′) CTTCGCGGGCGACGAT, Reverse (5′–3′) CCACATAGGAATCCTTCTGACC.

### 2.4 Western Blot

The protein was isolated using the Total Protein Extraction Kit (BC3710, Solarbio, Beijing, China). After mixing with the SDS‐PAGE Sample Loading Buffer (P0015, Beyotime), the samples were separated by electrophoresis on gels prepared with the SDS‐PAGE Gel Preparation Kit (P0012A, Beyotime). Following transfer to activated PVDF membrane (FFP26, Beyotime), the proteins were closed with 5% skim milk (BS102, Biosharp, Hefei, China). The incubation with anti‐WTAP (1:1000, ab195380, Abcam, Cambridge, UK), anti‐p53 (1:20000, 10442‐1‐AP, Proteintech, Wuhan, China), anti‐p16 (1:5000, 30519‐1‐AP, Proteintech), anti‐osteopontin (OPN) (1:3000, 22952‐1‐AP, Proteintech), anti‐osteocalcin (OCN) (1:5000, ab133612, Abcam), anti‐runt‐related transcription factor‐2 (RUNX2) (1:1000, 20700‐1‐AP, Proteintech), anti‐TP53BP1 (1:5000, ab175933, Abcam), and anti‐β‐actin (1:50000, 66009‐1‐Ig, Proteintech) as well as HRP‐conjugated Goat Anti‐Rabbit IgG (H + L) (1:10000, SA00001‐2, Proteintech) was conducted. Protein bands were visualized using the Amersham ImageQuant 800 Protein Blotting Imaging System (Cytiva, Psala, Sweden) and quantized with the Image J software (version 1.8.0).

### 2.5 Isolation and Identification of Periodontitis‐Derived PDLSCS (P‐PDLSCs)

PDL tissues were isolated from the gingival samples of three independent donors with periodontitis (age: 43.1 ± 9.5 years, *n* = 3), who provided written informed consent. Each donor sample was considered as an independent biological replicate. P‐PDLSCs were isolated and cultured as previous described [16]. Briefly, PDL tissues were minced and digested in type I collagenase (BS163, Biosharp) and dispase (BL1360A, Biosharp) at 37°C for 30 min. Following centrifugation at 37°C 1000 g for 5 min, the pellet was resuspended and cultured in the α‐MEM (OMDCM‐047, Oumarsi, Shanghai, China) added with 10% fetal bovine serum (FBS, A5256701, Gibco, Grand Island, NY, USA) and 1% penicillin/streptomycin (P/S, SV30010, G‐CLONE, Beijing, China). Primary P‐PDLSCs were observe on days 6 and 12. Upon reaching 90% confluence, cells were passaged, and experiments were carried out with the cells between passages 2 and 5.

### 2.6 Alkaline Phosphatase (ALP) Staining

P‐PDLSCs were spread into 6‐well plates and subjected to different treatments. Osteogenic differentiation was induced for 7 days using α‐MEM (OMDCM‐047, Oumarsi) containing 10% FBS (A5256701, Gibco), 1% P/S (SV30010, G‐CLONE), dexamethasone (0.1 μM, 40323ES03, Yeasen, Shanghai, China), β‐glycerophosphate (10 mM, ST637, Beyotime), and ascorbic acid (50 μM, 60374ES60, Yeasen). Then the cells were treated with 4% paraformaldehyde (PFA, P0099, Beyotime) for 10 min and with BCIP/NBT dyeing solution (C3206, Beyotime) for 30 min away from light. Staining intensity was quantified using the Image J 1.8.0 software.

### 2.7 Oil Red O Staining

P‐PDLSCs were induced toward adipogenic differentiation for 21 days using a Lipogenic induction differentiation medium (AW‐MY002, Abiowell, Changsha, China). After that, cells were treated with 4% PFA (P0099, Beyotime) for 30 min, and the staining with oil red O working solution (AWI0579a, Abiowell) was performed for 30 min. Stained lipid droplets were visualized under the microscope (Keyence, Osaka, Japan).

### 2.8 Flow Cytometry

P‐PDLSC suspensions were incubated with APC‐conjugated antibodies against CD45 (ab28106, Abcam), CD34 (ab310881, Abcam), CD105 (ab60902, Abcam), and PE‐conjugated anti‐CD90 (ab33694, Abcam) at 4°C away from light. Flow cytometry was conducted to identify stem cell markers.

Reactive oxygen species (ROS) levels were tested using the ROS Assay Kit (S0033, Beyotime). Teated P‐PDLSCs were reacted with DCFH‐DA at 37°C for 20 min, and results were analyzed by flow cytometry.

### 2.9 Vector Establishment and Transfection

Short hairpin (sh)‐RNAs targeting WTAP (shWTAP) and TP53BP1 (shTP53BP1), the overexpression vector for WTAP (oeWTAP, #53741, Addgene, Beijing, China), and corresponding negative controls (shNC and oeNC) were designed and synthesized by Tsingke (Beijing, China). Based on the specification of Lipo2000 (A5230‐025, BDBIO, Hangzhou, China), transfection was conducted as required. The efficiencies were verified by western blot.

### 2.10 Malondialdehyde (MDA) Measurement

MDA contents were tested by exploiting the Lipid Peroxidation MDA Assay Kit (S0131, Beyotime). Cells were cracked with Cell lysis buffer (P0013, Beyotime), and the supernatant was collected after centrifugation. Protein concentration was confirmed for normalization. The standard substance was prepared using MDA standards at 1, 2, 5, 10, 20, and 50 µM. Samples and standards were mixed with MDA detection working solution, heated at 100°C for 10 min, cooled, and centrifuged. Absorbance was tested at 532 nm, and results were determined according to the standard curve.

### 2.11 Superoxide Dismutase (SOD) Examination

SOD contents were examined applying the Total Superoxide Dismutase Assay Kit with WST‐8 (S0101, Beyotime). Briefly, cells were treated with the SOD sample preparation solution, and the supernatant was collected after centrifugation. Samples were treated with WST‐8/enzyme working solution and reaction start solution at 37°C for 30 min, and absorbance was tested at 450 nm. Result was calculated accordingly.

### 2.12 Immunofluorescence (IF)

Treated P‐PDLSCs were fixed and blocked with QuickBlock Blocking Buffer for Immunol Staining (P0260, Beyotime) for 15 min, followed by incubation with γ‐H2AX rabbit monoclonal antibody at 37°C for 1 h. Cells were reacted with the Anti‐rabbit 488 at 37°C for 1 h, and nuclei were treated with DAPI for 5 min. Images were acquired under the microscope (Keyence) and quantified with Image J 1.8.0 software. All steps were conducted employing the DNA Damage Assay Kit by γ‐H2AX Immunofluorescence (C2035, Beyotime).

### 2.13 Cell Senescence Detection

Based on the manual of the Senescence β‐Galactosidase Staining Kit (C0602, Beyotime), the senescence of cells was measured. After treatment, cells were treated with β‐galactosidase staining fixative for 15 min, then incubated with dye working solution prepared by β‐galactosidase stain solution A/B/C and X‐Gal solution overnight. Stained cells were observed using the microscope (Keyence) and quantified using the Image J 1.8.0 software.

### 2.14 Alizarin Red S (ARS) Staining

P‐PDLSCs were inoculated into the 6‐well plates and induced toward osteogenic differentiation for 28 days. Cells were treated with 4% PFA (P0099, Beyotime) for 10 min and with ARS staining solution (G1452, Solarbio) for 10 min, respectively. Mineralized nodules were visualized under the microscope (Keyence). The 10% cetylpyridinium chloride monohydrate (IK‐OIN‐5, Solarbio) was applied to dissolve the calcium deposits, and the absorbance at 562 nm was tested under the microplate reader (Molecular Devices, Shanghai, China). The Image J 1.8.0 software was used for additional quantification.

### 2.15 Methylated RNA Immunoprecipitation (MeRIP)

m6A methylation levels on TP53BP1 mRNA were analyzed utilizing the GenSeq m6A MeRIP Kit (GS‐ET‐001B, Cloud‐seq, Shanghai, China). RNA was fragmented to ≤ 200 n, and immunoprecipitation was performed using m6A antibody‐bound magnetic beads. Purified RNA was subjected to qPCR analysis.

### 2.16 RNA Immunoprecipitation (RIP)

RIP was carried out employing the BersinBio RIP Kit (Bes5101, BersinBio, Guangzhou, China). Cell lysates were prepared using polysome lysis buffer supplemented with Protease and RNase inhibitor. Lysates were incubated with protein A/G magnetic beads coupled with anti‐IgG or anti‐WTAP (1:40, ab195380, Abcam). Precipitated RNA was extracted and analyzed by qPCR.

### 2.17 mRNA Stability

P‐PDLSCs were treated with shNC or shWTAP in combination with Actinomycin D (Act D, HY‐17559, MCE, Shanghai, China) for 0, 2, 4, and 6 h. RNA was extracted, and TP53BP1 expression was assessed by qPCR using the 2^−^^ΔΔCt^ method.

### 2.18 Dual‐Luciferase Reporter Assay

Wild‐type (WT) and mutant (MUT) TP53BP1 3′UTR luciferase reporter plasmids (WT‐TP53BP1 and MUT‐TP53BP1) were constructed by Sangon Biotech (Shanghai) Co. Ltd. (Shanghai, China). Cells were co‐transfected with WT‐TP53BP1 or MUT‐TP53BP1 and shNC or shWTAP. After 24 h, luciferase activity was evaluated using the Dual Luciferase Reporter Gene Assay Kit (RG027, Beyotime).

### 2.19 Statistical Method

Experimental results are represented as the mean ± SD from at least three independent experiments. Data were evaluated applying GraphPad Prism 8.4.3 software (GraphPad Prism, San Diego, CA, USA). For comparisons between 2 independent groups, Unpaired *t* test was used. For 3 or more groups, one‐way ANOVA was applied. If ANOVA suggested significant differences, Tukey's post hoc test was used for multiple comparisons to control Type I errors. In cases where data did not meet normality assumptions, the Mann‐Whitney U test was conducted. *p* < 0.05 was deemed significant.

## 3 Results

### 3.1 WTAP Is Upregulated in Periodontitis Cells and Tissues

**Figure 1:**
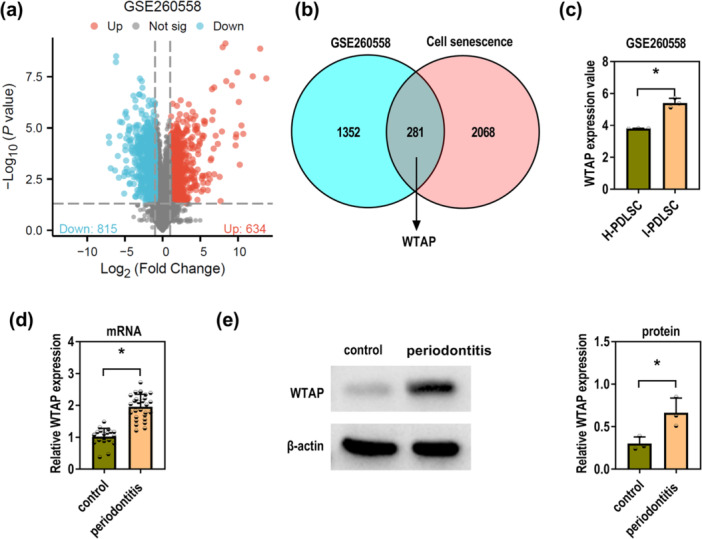
WTAP expression is elevated in periodontitis. (a) The volcano plot showed the gene expression profile in the GSE260558 dataset. (b) The Venn diagram showed the intersection of the DEGs from the GSE260558 dataset and the cell senescence‐related genes from the Genecards database. (c) WTAP expression in H‐PDLSCs and I‐PDLSCs of the GSE260558 dataset (Welch's *t*‐test). (d) RT‐qPCR was used to detect WTAP mRNA levels in control (*N* = 20) and periodontitis (*N* = 28) tissues (Unpaired *t* test). (e) Western blot was used to detect WTAP protein levels in control and periodontitis tissues (Unpaired *t* test). All experiments were conducted at least three independent times. *p < 0.05.

To investigate DEGs in I‐PDLSCs, the GSE260558 dataset was analyzed using the GEO2R tool. This analysis identified 815 downregulated and 634 upregulated genes (Figure 1a). These DEGs were then cross‐referenced with cell senescence‐related genes from the Genecards database (relevance score ≥ 10), revealing 281 overlapping genes, including WTAP (Figure 1b). WTAP expression was significantly higher in I‐PDLSCs than in H‐PDLSCs (Figure 1c). Besides, WTAP expression in clinical samples was validated using RT‐qPCR and western blot. Both assays confirmed that WTAP expression were elevated in periodontitis tissues compared to normal gingival tissues (Figure 1d,e). Analysis of baseline clinical characteristics showed no significant differences in gender, age, or smoking status between periodontitis patients and the control group. However, periodontitis patients exhibited worse periodontal conditions across all clinical parameters assessed, including mean maximum probing depth per tooth, clinical attachment loss, bleeding on probing, and plaque index. Furthermore, all periodontitis patients were diagnosed with stage III disease of severity grade B or C, underscoring the advanced nature of the periodontal pathology (Table 1). More importantly, a significant positive correlation was observed between WTAP expression levels in periodontitis gingival tissues and mean maximum probing depth per tooth (Figure S1). Therefore, these results demonstrate that WTAP is highly expressed in periodontitis and suggest its potential involvement in the pathogenesis of the disease.

**Table 1:** Clinical features of the patients with periodontitis and control.

	Control (*n* = 20)	Periodontitis (*n* = 28)	*p* value
Males/females	11/9	16/12	**0.883**
Age (years)	45.6 ± 8.3	43.1 ± 9.5	**0.349**
Smoking status % (*n*)	15.0(3)	21.4(6)	**0.851**
Mean maximum probing depth per tooth (mm)	2.0 ± 0.4	5.8 ± 0.8	**< 0.001**
Clinical attachment loss (mm)	0.0 ± 0.0	4.9 ± 1.6	**< 0.001**
Bleeding on probing (% of sites)	8.2 ± 1.7	79.2 ± 10.6	**< 0.001**
Plaque Index (0–3)	0.6 ± 0.2	2.2 ± 0.5	**< 0.001**
Disease Stage (III/IV, *n*)	—	28/0	
Disease Grade (B/C, *n*)	—	15/13	

*Note:* Continuous variables are presented as Mean ± Standard Deviation and were compared using the Independent Samples *t*‐test. Categorical variables are presented as number (*n*) and percentage (%) and were compared using the Chi‐square test or Fisher's exact test. Periodontitis diagnosis: All patients in the periodontitis group were diagnosed according to the 2018 World Workshop on the Classification of Periodontal and Peri‐Implant Diseases and Conditions. The inclusion criteria required the presence of at least two non‐adjacent teeth with Probing Depth ≥ 5 mm, Clinical Attachment Loss ≥ 3 mm, and radiographic evidence of alveolar bone loss exceeding 15% of the root length. Healthy Control Criteria: Subjects in the healthy control group had no history of periodontitis, with full‐mouth Probing Depth ≤ 3 mm, no Clinical Attachment Loss, and Bleeding on Probing at < 10% of sites. *p* < 0.05 was considered statistically significant and was indicated in bold.

### 3.2 The Characterization of P‐PDLSCs

**Figure 2:**
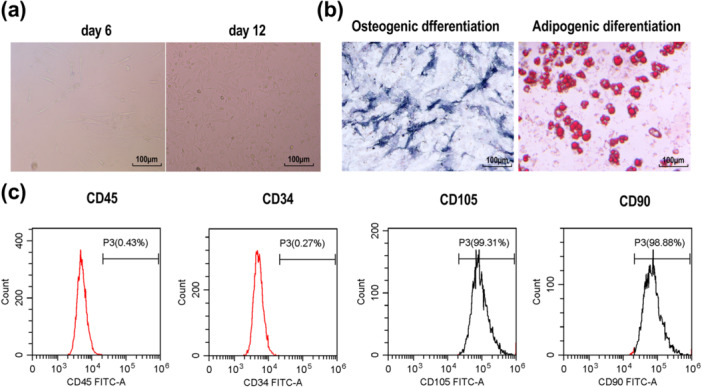
P‐PDLSC separation and identification. (a) Morphology of primary P‐PDLSCs observed by microscope (Scale bar = 100 μM). (b) The osteogenic and adipogenic differentiation potential of P‐PDLSCs were detected using ALP staining and oil red O, respectively (Scale bar = 100 μM). (c) Flow cytometry analysis of P‐PDLSCs.

Primary P‐PDLSCs typically exhibited a spindle‐shaped or fibroblast‐like morphology (Figure 2a). These cells demonstrated normal multilineage differentiation potential, including the ability to undergo osteogenic and adipogenic differentiation (Figure 2b). In addition, flow cytometry analysis confirmed that the isolated P‐PDLSCs expressed high levels of the mesenchymal stem cell (MSC) markers (CD105 and CD90), while showing low expression of hematopoietic cell markers (CD45 and CD34) (Figure 2c). These results indicate that the isolated P‐PDLSCs are MSCs with osteogenic and lipogenic potential.

### 3.3 WTAP Knockdown Inhibits P‐PDLSC Senescence and Oxidative Stress

**Figure 3:**
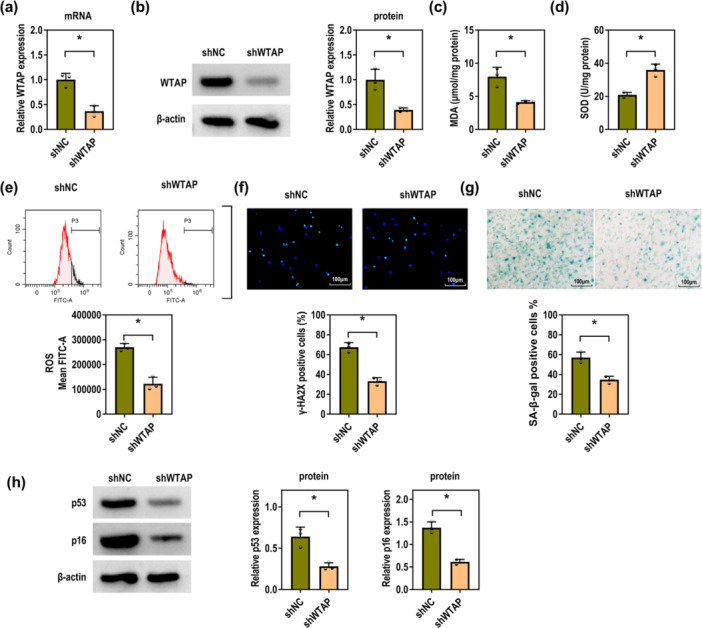
WTAP downregulation restrains P‐PDLSC senescence and oxidative stress. The P‐PDLSCs were transfected with shNC and shWTAP. (a) RT‐qPCR was used to measure the mRNA levels of WTAP (Unpaired *t*‐test). (b) Western blot was applied to determine the protein levels of WTAP (Unpaired *t*‐test). (c–e) The MDA, SOD, and ROS levels were examined using the corresponding kits (Unpaired *t* test). (f) The γ‐H2AX positive cells were detected using IF (Scale bar = 100 μM; Unpaired *t*‐test). (g) SA‐β‐gal positive cells were measured using the corresponding kit (Scale bar = 100 μM; Unpaired *t* test). (h) Western blot was applied to determine the protein levels of p53 and p16 (Unpaired *t*‐test). All experiments were conducted at least three independent times. *p < 0.05.

To explore the effects of WTAP, P‐PDLSCs were transfected with shWTAP. Compared to the shNC group, WTAP was significantly downregulated at both mRNA and protein levels in the shWTAP group (Figure 3a,b). Knockdown of WTAP led to a decrease in MDA levels and an increase in SOD activity (Figure 3c,d). Furthermore, the ROS levels were decreased in shWTAP‐transfected P‐PDLSCs compared with the shNC group (Figure 3e). Meanwhile, the IF results displayed that WTAP knockdown decreased the number of γ‐H2AX positive cells, indicating a reduction in DNA damage (Figure 3f). The number of SA‐β‐gal positive cells was also decreased upon WTAP silencing (Figure 3g). Consistently, the expression levels of senescence‐related proteins p53 and p16 were notably downregulated in shWTAP‐transfected P‐PDLSCs (Figure 3h). Collectively, silencing WTAP represses the senescence and oxidative stress in P‐PDLSCs.

### 3.4 Silencing WTAP Promotes P‐PDLSC Osteogenic Differentiation

**Figure 4:**
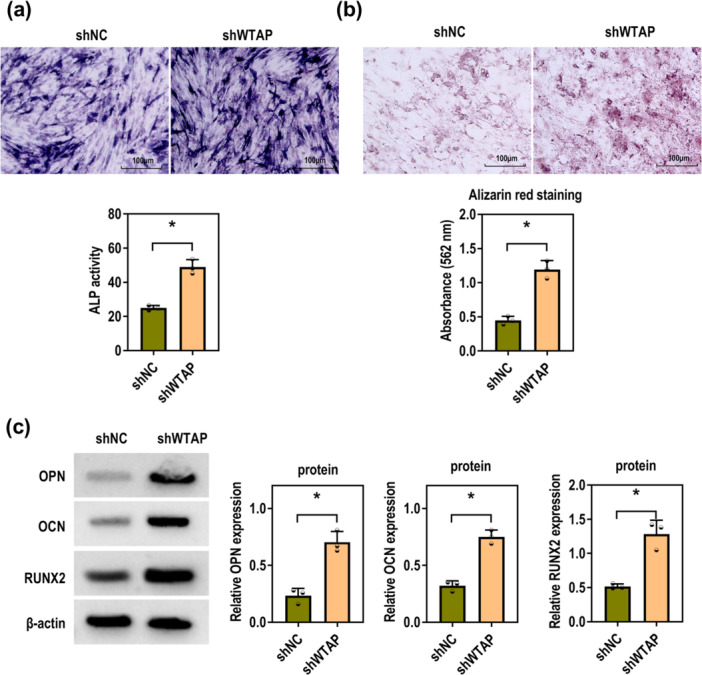
WTAP downregulation facilitates P‐PDLSC osteogenic differentiation. The P‐PDLSCs were transfected with shNC and shWTAP. (a) ALP staining of P‐PDLSCs induced for 7 days (Scale bar = 100 μM; Unpaired *t* test). (b) ARS staining of P‐PDLSCs induced for 28 days (Scale bar = 100 μM; Unpaired *t*‐test). (c) Western blot was used to determine the protein levels of OPN, OCN, and RUNX2 (Unpaired *t* test). All experiments were conducted at least three independent times. *p < 0.05.

As shown in Figure 4a,b, knockdown of WTAP enhanced the osteogenic differentiation capacity of P‐PDLSCs, as evidenced by increased ALP activity and more abundant calcium nodule formation. Additionally, OPN, OCN, and RUNX2, the osteogenic differentiation‐related proteins, were increased by WTAP downregulation (Figure 4c). Consequently, WTAP knockdown accelerates the osteogenic differentiation of P‐PDLSCs.

### 3.5 WTAP Promotes TP53BP1 Expression through m6A Modification

**Figure 5:**
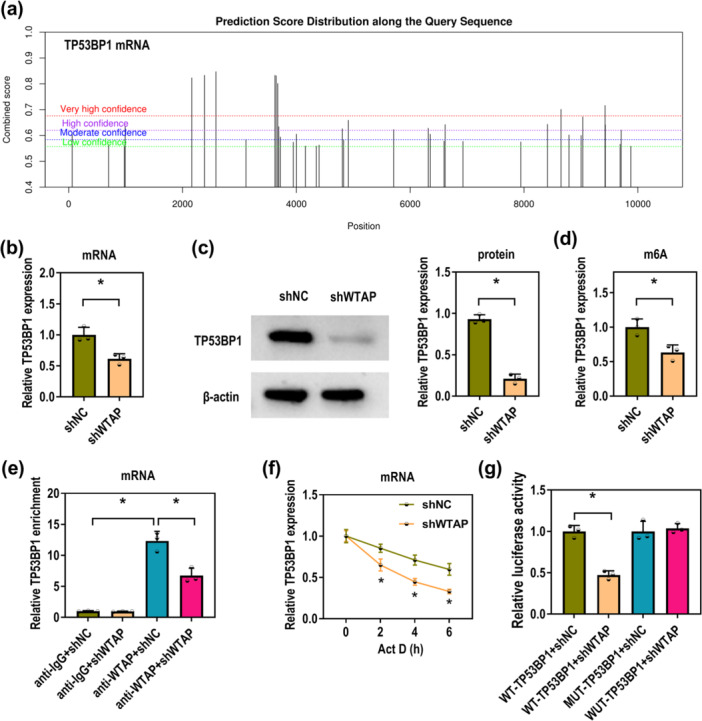
WTAP modifies TP53BP1 via m6A. (a) The m6A sites of TP53BP1 were predicted by the SRAMP website. (b, c) TP53BP1 expression in P‐PDLSCs transfected with shNC and shWTAP was measured by RT‐qPCR and western blot, respectively (Unpaired *t*‐test). (d) MeRIP was used to determine the m6A levels on TP53BP1 mRNA in P‐PDLSCs transfected with shNC and shWTAP (Unpaired *t*‐test). (e) TP53BP1 mRNA enrichment in P‐PDLSCs treated with anti‐IgG + shNC, anti‐IgG + shWTAP, anti‐WTAP+shNC, and anti‐WTAP + shWTAP was examined by RIP (one‐way ANOVA). (f) TP53BP1 mRNA expression in P‐PDLSCs co‐treated with shNC or shWTAP and Act D for 0, 2, 4, and 6 h was detected by RT‐qPCR (one‐way ANOVA). (g) Luciferase activities in the WT‐TP53BP1 + shNC, WT‐TP53BP1 + shWTAP, MUT‐TP53BP1 + shNC, and MUT‐TP53BP1+shWTAP groups were measured by dual‐luciferase reporter assay (one‐way ANOVA). All experiments were conducted at least three independent times. *p < 0.05.

To investigate the mechanism of WTAP in P‐PDLSCs, the subsequent experiments were further conducted. Given that TP53BP1 has been reported to promote senescence in PDLSCs [15], it is hypothesized that TP53BP1 may act as a downstream target of WTAP in periodontitis. Using the SRAMP database, this study identified potential m6A modification sites in TP53BP1 mRNA (Figure 5a). Besides, WTAP knockdown led to a reduction in TP53BP1 expression at both mRNA and protein levels (Figure 5b,c). Meanwhile, MeRIP assay showed that silencing WTAP reduced the m6A levels on TP53BP1 mRNA (Figure 5d). Also, RIP results further confirmed the enrichment of TP53BP1 by WTAP antibody, and this enrichment was reduced in the anti‐WTAP+shWTAP group compared to the anti‐WTAP+shNC group (Figure 5e). As described in Figure 5f, TP53BP1 mRNA stability was reduced upon WTAP knockdown, with a faster decline in expression after Act D treatment. Further, dual‐luciferase reporter assay revealed that WTAP knockdown decreased the luciferase activity in the WT‐TP53BP1 group, but not in the MUT‐TP53BP1 group (Figure 5g). Accordingly, WTAP enhances TP53BP1 expression through m6A‐dependent mRNA regulation.

### 3.6 Silencing TP53BP1 Undermines WTAP‐Mediated Senescence and Oxidative Stress of P‐PDLSCs

**Figure 6:**
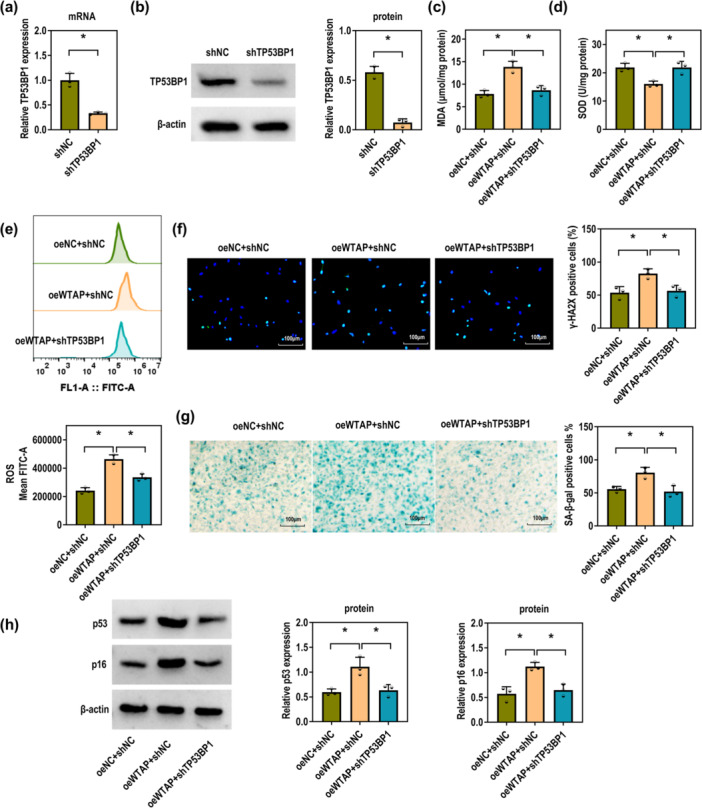
TP53BP1 downregulation mitigates WTAP‐caused P‐PDLSC senescence and oxidative stress. (a) RT‐qPCR was used to measure the mRNA levels of TP53BP1 in P‐PDLSCs transfected with shNC and shTP53BP1 (Unpaired *t*‐test). (b) Western blot was applied to determine the protein levels of TP53BP1 in P‐PDLSCs transfected with shNC and shTP53BP1 (Unpaired *t*‐test). (c–h) The P‐PDLSCs were treated with oeNC + shNC, oeWTAP + shNC, and oeWTAP + shTP53BP1. (c–e) The MDA, SOD, and ROS levels were examined using the corresponding kits (one‐way ANOVA). (f) The γ‐H2AX positive cells were detected using IF (Scale bar = 100 μm; one‐way ANOVA). (g) SA‐β‐gal positive cells were measured using the corresponding kit (Scale bar = 100 μM; one‐way ANOVA). (h) Western blot was applied to determine the protein levels of p53 and p16 (one‐way ANOVA). All experiments were conducted at least three independent times. *p < 0.05.

Transfection with shTP53BP1 effectively downregulated TP53BP1 expression in P‐PDLSCs compared to the shNC group (Figure 6a,b). The increases in MDA and ROS levels, as well as the decrease in SOD activity induced by WTAP overexpression, were attenuated by TP53BP1 knockdown (Figure 6c–e). Similarly, the elevated numbers of γ‐H2AX and SA‐β‐gal positive cells caused by WTAP overexpression were reduced upon TP53BP1 silencing (Figure 6f,g). Furthermore, silencing of TP53BP1 reversed WTAP‐enhanced expression of p53 and p16 in P‐PDLSCs (Figure 6h). As such, TP53BP1 knockdown ameliorates the senescence and oxidative stress of P‐PDLSCs caused by WTAP overexpression.

### 3.7 WTAP Impedes P‐PDLSC Osteogenic Differentiation by Accelerating TP53BP1 Expression

**Figure 7:**
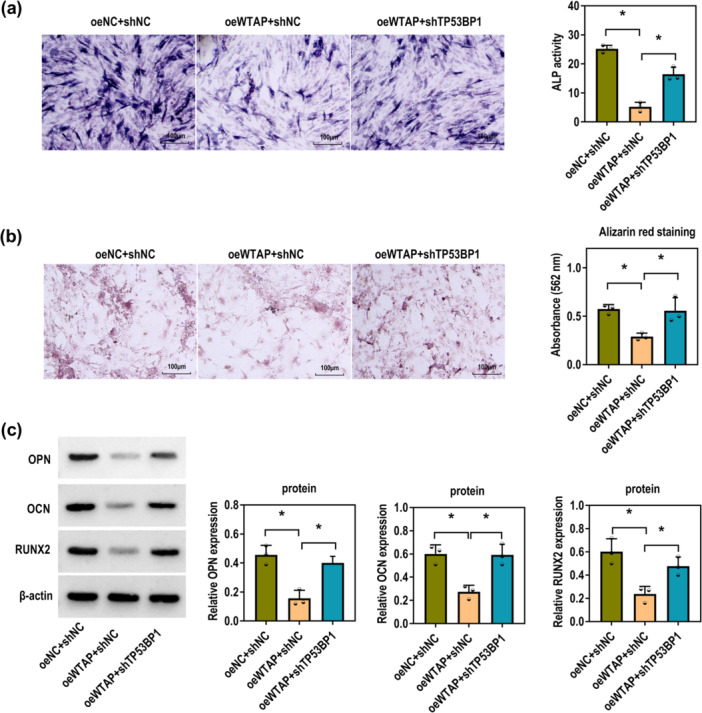
WTAP represses the osteogenic differentiation of P‐PDLSCs via TP53BP1. The P‐PDLSCs were treated with oeNC+shNC, oeWTAP+shNC, and oeWTAP + shTP53BP1. (a) ALP staining of P‐PDLSCs induced for 7 days (Scale bar = 100 μM; one‐way ANOVA). (b) ARS staining of P‐PDLSCs induced for 28 days (Scale bar = 100 μM; one‐way ANOVA). (c) Western blot was used to determine the protein levels of OPN, OCN, and RUNX2 (one‐way ANOVA). All experiments were conducted at least three independent times. *p < 0.05.

The inhibitory effect of WTAP overexpression on osteogenic differentiation was rescued after TP53BP1 knockdown, as demonstrated by enhanced ALP activity and mineralized nodule formation (Figure 7a,b). Consistently, the downregulation of OPN, OCN, and RUNX2 expression induced by WTAP overexpression was reversed upon TP53BP1 silencing (Figure 7c). The above results suggest that WTAP inhibits osteogenic differentiation of P‐PDLSCs by promoting TP53BP1 expression.

## 4 Discussion

Periodontitis, a chronic inflammatory disease, has been increasingly linked to an elevated risk of various systemic conditions, such as cardiovascular disease, diabetes, and even malignancies such as oral cancer [17, 18, 19]. Given its impact on both oral and systemic health, elucidating the pathogenesis of periodontitis and exploring its molecular targets are particularly important for the prevention and treatment of periodontitis. PDLSCs, cells with self‐renewal and multi‐directional differentiation potentials, are indispensable in promoting the repair and regeneration of periodontal tissues [20]. A previous study in porcine periodontitis has confirmed that when PDLSCs are transplanted into the periodontal defect area, regenerated periodontal tissues could be obtained [21]. However, with the increase of age or stimulation, PDLSCs will experience functional disorders and aging, thereby leading to a decline in their biological characteristics and functions [15].

Herein, this study identified WTAP as being significantly upregulated in periodontitis tissues and P‐PDLSCs. This finding positions WTAP as a responsive element to the inflammatory milieu of periodontitis. Chronic exposure to pro‐inflammatory cytokines like TNF‐α and IL‐1β, hallmarks of the periodontitis microenvironment [22], is a likely driver of this elevated WTAP expression. This aligns with the emerging concept that the inflammatory landscape can reshape the epitranscriptome to perpetuate disease states. Therefore, these data suggest that WTAP upregulation is a maladaptive response to inflammation, which in turn orchestrates the downstream pathological events in P‐PDLSCs. Meanwhile, WTAP expression levels may serve as a novel molecular biomarker. Future applications could involve measuring WTAP levels in gingival crevicular fluid or saliva, or performing WTAP immunohistochemical staining on periodontal tissue biopsy samples to aid in the diagnosis, severity grading, or activity assessment of periodontitis. When combined with traditional clinical indicators (such as probing depth and bleeding index), this approach may provide more objective molecular‐level evidence.

The core pathological event this study investigated was PDLSC senescence, a key mechanism undermining periodontal regeneration [23]. This research demonstrated that knockdown of WTAP markedly alleviated senescence and associated oxidative stress in P‐PDLSCs. This anti‐senescence effect was mechanistically rooted in the WTAP/TP53BP1 axis. TP53BP1 is a well‐established DNA damage response protein [14, 24], and its role in promoting PDLSC senescence in inflammation has been recently highlighted [15]. This study significantly extends this understanding by identifying TP53BP1 as an m6A target. It was proved that WTAP stabilized TP53BP1 mRNA via m6A modification, leading to its accumulation. This creates a feed‐forward loop where in inflammation‐induced WTAP elevates TP53BP1, which amplifies the DNA damage signal and drives cells into an irreversible senescent state. This m6A‐dependent stabilization of TP53BP1 provides a novel molecular explanation for the sustained senescence phenotype in P‐PDLSCs under inflammatory stress, complementing the known regulation via alternative splicing [15].

A critical consequence of P‐PDLSC senescence is the failure to regenerate alveolar bone [23]. Consistent with this, it was found that the WTAP/TP53BP1 axis acted as a potent brake on osteogenic differentiation. The rescue of osteogenic potential upon TP53BP1 knockdown, even in the context of WTAP overexpression, unequivocally linked the pro‐senescence function of this axis to the impaired regeneration. This inverse relationship between senescence activation and osteogenic differentiation is a pivotal concept in periodontitis pathogenesis [25]. This work delineates a unified pathway—WTAP‐mediated m6A modification of TP53BP1—that simultaneously accelerates senescence and inhibits osteogenesis, thereby directly contributing to the net loss of periodontal bone. However, these studies diverge significantly in the proposed cellular mechanisms and downstream consequences. Li *et al*. focused on the role of WTAP in macrophage M1 polarization and reported that downregulation of WTAP boosted the osteogenic differentiation of bone marrow stromal cells [13]. In contrast, this research elucidated a direct intrinsic mechanism in P‐PDLSCs, where by WTAP promoted cellular senescence and inhibited osteogenic differentiation by stabilizing TP53BP1 mRNA. This discrepancy highlights the cell‐type‐specific functions of WTAP within the complex periodontal microenvironment. It is plausible that WTAP exerts distinct and perhaps opposing roles in immune cells versus stem cells, fine‐tuning the balance between inflammation and regeneration in periodontitis. This work complements that of Li *et al*. by providing a mechanistic explanation for the impaired regenerative capacity of P‐PDLSCs, suggesting that targeting the WTAP/TP53BP1 axis could preserve stem cell function independently of its immunomodulatory effects. More importantly, this provides new insights for developing targeted therapeutic strategies. For instance, small‐molecule inhibitors or shRNA therapies targeting WTAP could theoretically disrupt this pathogenic pathway, thereby alleviating P‐PDLSC senescence, promoting their osteogenic differentiation, and ultimately achieving periodontal tissue regeneration and repair. Although m6A‐targeted therapeutics remains in the early stages of research, this work points to a concrete direction for epigenetic treatment of periodontitis. Furthermore, given TP53BP1's central role in DNA damage response, targeting this axis may also improve genomic stability within the periodontitis microenvironment, offering new insights for combination therapies.

To sum up, this study delineates a novel epigenetic pathway driving periodontitis. It is identified that WTAP is significantly upregulated in periodontitis, and its knockdown alleviates cellular senescence and oxidative stress while enhancing the osteogenic potential of P‐PDLSCs. Mechanistically, this research demonstrates that WTAP directly binds to and stabilizes TP53BP1 mRNA via m6A modification, thereby establishing the WTAP/TP53BP1 axis as a key regulator of PDLSC dysfunction in an m6A‐dependent manner.

While these *in vitro* findings provide compelling evidence, they also pave the way for several critical future investigations. First, the most immediate next step is *in vivo* validation using animal models of periodontitis. Testing the efficacy of locally administered WTAP or TP53BP1 inhibitors in preventing alveolar bone loss would be a crucial translation of these findings. Second, exploring the therapeutic implications by screening for small‐molecule inhibitors or developing targeted nanocarriers for siRNA delivery against this axis holds significant promise for a novel host‐modulation therapy. Finally, investigating whether WTAP and TP53BP1 expression levels can stratify patients with periodontitis (such as predicting disease aggressiveness or response to regeneration therapies) would explore their utility as prognostic biomarkers.

Collectively, this study not only expands the understanding of the epigenetic landscape of periodontitis but also nominates the WTAP/TP53BP1 axis as a compelling and viable target for future diagnostic and therapeutic development.

## Author Contributions

Tingting Wang designed and performed the research. Yuan Liu analyzed the data. Menglin Xiong wrote the manuscript. All authors read and approved the final manuscript.

## Funding

The authors received no specific funding for this work.

## Ethics Statement

Written informed consents were obtained from all participants and this study was permitted by the Ethics Committee of the School/Hospital of Stomatology, The First Affiliated Hospital of Xinjiang Medical University.

## Conflicts of Interest

The authors declare no conflicts of interest.

## Supporting information


**Supplementary Figure 1:** The positive correlation between WTAP expression levels in periodontitis gingival tissues and mean maximum probing depth per tooth.

## Data Availability

The analyzed data sets generated during the present study are available from the corresponding author on reasonable request.
