# Supplementary figures and images for: WTAP Contributes to Periodontitis Pathogenesis by Promoting PDLSC Senescence and Impairing Osteogenic Differentiation via m6A‐Dependent Regulation of TP53BP1
Source: Immun Inflamm Dis. 2026 Feb 5;14(2):e70335. doi: 10.1002/iid3.70335 (PMC12873629; doi:10.1002/iid3.70335)

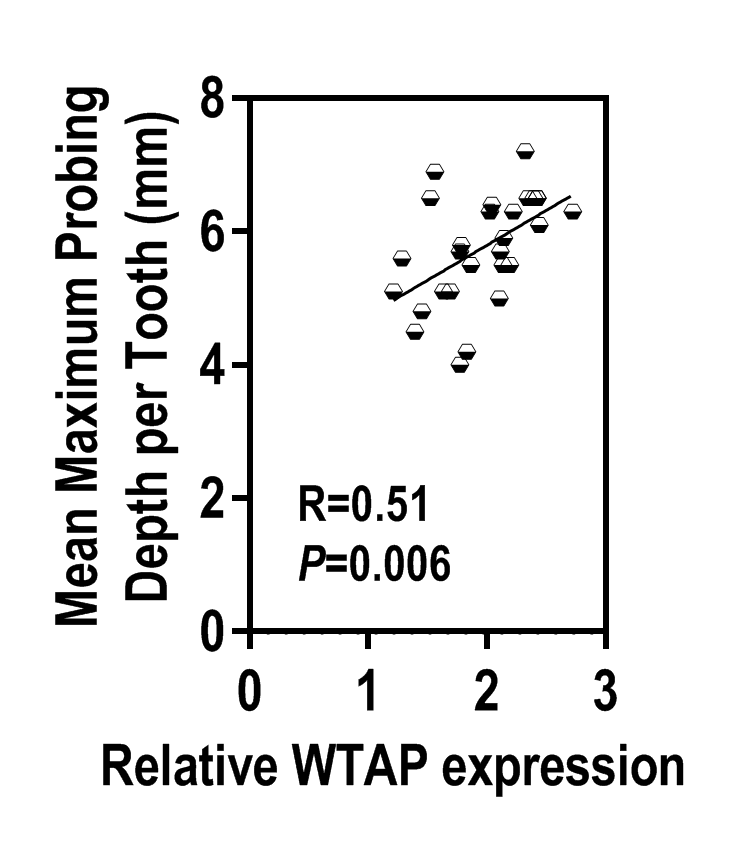

Supplement: Supplementary file 1 — Supplementary Figure 1: The positive correlation between WTAP expression levels in periodontitis gingival tissues and mean maximum probing depth per tooth. [file IID3-14-e70335-s001.tif]
